# Suppurative Labyrinthitis

**Published:** 1912-07

**Authors:** Chester C. Cott

**Affiliations:** Buffalo, N. Y.; 1195 Main Street


### Suppurative Labyrinthitis

By CHESTER C. COTT, M.D.
Buffalo, N. Y.

ACCORDING to statistics there are two cases of suppurative labyrinthitis to every one hundred of middle ear suppuration. Therefore in a city the size of ours there must be several cases every year. It is only a few years ago that the diagnosis was first made so the classical symptoms of the disease have not reached all physicians. Consequently this disease has, up to this time, not been commonly diagnosed so I shall take this opportunity of describing it to you.

Purulent involvement of the labyrinth may occur from within the skull or from the middle ear. The most frequent intracranial cause is cerebro-spinal meningitis. The infection advances up the internal auditory meatus and results in an empyæma of the labyrinth. If the patient recovers, bilateral deafness is present. Most of the inmates of our deaf-mute asylums have been made deaf by this means. There is no help for the patient in the course of the disease. However when the infection goes from the middle ear to the labyrinth, usually thro the round or oval window, assistance can be given. Such is the path in scarlet fever and measles. Middle ear suppuration complicating these two diseases is most liable to enter the labyrinth and it is this form which I shall describe tonight.

In order to make its couse clear the anatomy of the ear must be understood. You remember that the auditory apparatus is divided into three parts, the external, middle and internal ear. The external ear is composed of auricle, the cartilaginous and bony meatus to the drumhead. The middle ear includes the mastoid antrum, the tympanic cavity and its contents and the eustachian tube. The tympanic cavity is shut in externally by the drumhead, and internally is separated from the inner ear by a thin wall of bone which has two openings, into one of which the footplate of the stapes is fastened, while the other is closed by membrane only. The internal ear or labyrinth is situated in the petrous portion of the temporal bone surrounded on all sides by thin compact bone, which separates it externally from the middle ear, anteriorly from the middle fossa, and posteriorly from the posterior fossa.

The labyrinth is a complicated system of bony canals. In the center is the vestibule, a small cavity into which the three semicircular canals, lying posteriorly open. The cochlea lies anterior to the vestibule and also opens into it. A layer of endothelium lines the bony labyrinth. Inside this system of canals is a similar system of membranous canals, which are about 1-5 the size of the former. In the space which remains there is a fluid called perilymph whichsurrounds the membranous canals on nearly all sides and communicates with the subdural space. Inside the *membranous* labyrinth there is also a fluid which is called endolymph. This has no outlet except a blind sac which lies upon the dura of the posterior fossa.

Altho the inner ear is one system, it has three functions: that of hearing which is done by the cochlea: that of interpreting turning motions of the head which is done by the semicircular canals; and that of perceiving the position in which the head is held, which is done by two membranous sacs in the vestibule.

An exact knowledge of the condition of the inner ear can be obtained by the various tests at our command. The first point is to find out the state of hearing. It is important to find out whether an ear is absolutely deaf of not. If we find that it is, it may mean that the nerve ends in the membranous cochlea are destroyed by suppuration.

It is not a simple matter to find out whether an ear is totally deaf or not. It is difficult absolutely to exclude the other ear. The usual method is the use of some kind of noise-producing apparatus in the healthy ear and then the diseased ear is tested by talking or shouting if necessary. This is a crude way as the noise which obliterates hearing in the good ear may exclude a slight degree of hearing in the tested ear. This is overcome by Neuman's electric noise-producer, which conveys the noise produced in another room to the ear by wires. An equally good way is to allow a steady stream of water to run into the healthy ear and at the same time examine the other for hearing. This effectually deafens the good ear and causes no change in the examined ear. The six foot speaking tube test is also used. If an ear is totally deaf and the other not affected, the patient will answer the conversational voice three feet from the deaf ear when the other is held shut with the moistened finger. Now if under the same conditions a speaking tube of two meters length be used the patient will answer perfectly the conversational voice if any hearing for voice remains. These tests are always used together as one alone is not considered conclusive evidence.

We learn a great deal about the labyrinth from its characteristic nystagmus, which is different from any other kind of eye motions. It is characterized by a slow movement of the eyes in one direction, then a quick jerk toward the opposite side. It is always increased on looking in the direction of the quick component and decreased or stopped on looking toward the opposite side. It is usually rotatory, persists even when the eyes are closed, and gets milder upon repeated examinations. These points will differentiate labyrinthine nystagmus from any other kind. Labyrinthine nystagmus, if marked, is accompanied by a peculiar form of dizziness. The patient sees objects turn to the right or to the left or imagines that he is turning in the opposite direction. The attacks are always most marked upon arising in the morning and may be accompanied by vomiting. Of course at this time the patient's equilibrium will be disturbed. This same nystagmus which when present spontaneously is caused by disease can be produced by our tests.

In examining the semicircular canals all degrees of disease, whether degenerative, inflammatory or suppurative, may be ascertained. The most delicate test is this: a revolving chair that neither rises nor lowers is used. The left ear is tested by turning ten times to the right within ten seconds. If the ear is normal there will result a nystagmus to the left which will last about twenty-two seconds. The right ear is examined by turning to the left. A variation of the time of the persistence of the nystagmus present after turning tells us whether the canals are hypersensitive due to some focus of irritation or are less so or do not react at all.

The next most delicate test is the caloric or hot and cold water test. Cold water is allowed to run into the auditory meatus and normally after a few minutes nystagmus to the opposite side will appear. If hot water is used nystagmus to the same side is present. This result may appear quicker or later than normal or may not appear at all, in which case the labyrinth may be full of pus.

The mechanical test or Alexander's fistula symptom is performed by compressing and rarifying the air in the external auditory canal, which will cause a similar condition in the labyrinth if there is a fistula present. Then upon pressure there will be nystagmus to the opposite side. This is the usual sign and is characteristic of fistual into the horizontal semicircular canal.

The test by galvanic current is not so delicate since as long as the auditory nerve is alive it reacts. The test is best made by placing the poles on the ears. A current of about 4 milliamperes will normally cause a nystagmus toward the cathode.

The patient's equilibrium is tested by noting the difference between that maintained by the patient with eyes open and that with eyes closed. If there is no difference when standing on both feet with heels and toes together, standing on one foot is tried. By means of all these tests together an accurate and positive diagnosis of the labyrinth condition can usually be made.

One can easily imagine the advancing line of demarcation between the diseased and healthy bone in the middle ear and its walls. It gradually spreads until it involves the round or oval window, both of which open into the labyrinth. When the disease gets to this points involvement of the labyrinth begins. If the bony capsule of the labyrinth alone is diseased, it is called paralabyrinthitis. If the bone is necrosed down to the endothelial lining of the bony canal it is paralabyrinthitis with fistula. When this endothelium is destroyed involvement of the fluid in the bony canals or perilymph occurs, so it called perilabryinthitis. Finally the membranous canals and their contents the endolymph succumb and we have an endolabyrinthitis.

Paralabyrinthitis or diseased bony capsule of the labyrinth causes no symptoms by which we may recognize it before operation. There may be granulations or a polypus growing on the promontory or inner wall of the middle ear which would show that the capsule there is diseased but unless we can see signs of the bone disease, we cannot make a diagnosis of it. Still it is very often present in chronic middle ear suppuration.

When the caries extends thro the bony capsule and thus forms a fistula to the enothelial lining, the symptom of its presence appears, that is the nystagmus caused by raising and lowering the pressure in the external auditory canal, which does the same thing to the labyrinth thro the fistula. In addition upon testing there may be a slightly quicker appearance of the nystagmus, showing that the inner ear is more irritable than normal.

When the endolium lining the bony canal gives way and the infected material enters the perilymph there are some additional signs. Hearing is somewhat diminished. There is charasteristic dizziness with nystagmus toward the affected side. When doing the turning test the patient may become so dizzy after one or two turns that he will vomit. The caloric test will have the same result, the nystagmus appearing much quicker than normal or he may not be able to stand the test at all. Care must be taken in these cases to differentiate the spontaneous nystagmus from that which is caused by our tests. In this stage of the disease if the initial lesion was in a semicircular canal the trouble may remain local for some time but if the perforation was into the vestibule or cochlea it immediately becomes a diffuse perilabyrinthitis.

The next and final step is the succumbing of the membranous labyrinth and its contents the endolymph and nerve ends. With the rupture of this membrane the process immediately becomes general, that is the whole membranous labyrinth is attacked at once since it is simply a number of tubes which intercommunicate. With the entrance of pus into this system the inner ear *suddenly* ceases to perform its functions. There is sudden complete deafness in that ear, severe dizziness, perhaps vomiting, nystagmus toward the well side in all positions of the eyeball and disturbed equilibrium. The after-turning nystagmus caused by the affected ear will be practically negative, only a few twitches of the eyeball appearing. The caloric test will give no result, which shows that the labyrinth does not perform its function. The fistula symptom will be negative whether there is a fistula or not. The symptoms present remain severe for a short time only. After a few days they gradually become milder and disappear almost completely at the end of a week or ten days, if the patient survives. The spontaneous nystagmus will be present only on looking toward the healthy side and the equilibrium will be found to be slightly disturbed upon testing for it, but on the other tests the labyrinth will be found to be functionless.

Such is the cause of a purulent infection of the labyrinth. It may advance slowly or rapidly but the change from peri- to endolabyrinthitis is always sudden. At this time the nystagmus which has been toward the diseased side upon looking in that direction will suddenly change toward the well side and will be present no matter in which direction the patient looks. Severe dizziness with vomiting and sudden complete deafness occur at this time.

If this advancing involvement of the labyrinth is recognized in time, the hearing, perhaps the life, of the patient may be saved. The radical mastoid operation will usually cure all of these stages except the last one, suppurative endolabyrinthitis. When the pus has entered the membranous canals the radical no longer suffices. The labyrinth must be drained. If left alone the pus may break externally and heal spontaneously or it may rupture into the middle or posterior fossa causing extradural abscess, meningitis, encephalitis, brain abscess, etc. None of these results must occur but they very likely will. Therefore it is better to make a large external opening in a case of empyæma of the labyrinth and to remove all dead bone too, if the suppurative process of the labyrinth is chronic. Many other diseases of the labyrinth or auditory nerve have some symptoms which are similar to those described in this paper. All can be differentiated from the subject under discussion except possibly a serous inflammation of the labyrinth or labyrinthitis simplex. At a given time the patient may show signs and symptoms which will be applicable to both diseases In such a case there is usually something in the history which will point to one or the other disease. The serous inflammation practically always follows a traumatism, the ear usually does not become absolutely deaf, there is short nystagmus toward diseased side, and healing occurs within a few days or a week with persisting function of the auditory nerve, a result which is extremely rare after a suppurative involvement. The labyrinth is functionless only for the second and third and possibly fourth day of its seven days course. The differential diagnosis of these two conditions is most important as the treatment is so different.

Serous labyrinthitis should be left strictly alone while the suppurative inflammation must be operated.

In this paper I have given only the main points upon which a diagnosis of suppurative labyrinthitis is made. There are degenerative and toxic conditions of the nerve, arterio-sclerosis, syphilis, and other diseases which affect one or both branches of the eighth nerve, and cause signs and symptoms somewhat similar to those related here. The different diagnosis can easily be made. However time will not permit me to enter upon this subject. The diagnosis of suppurative labyrinthitis alone is sufficient for one evening's study.

1195 Main Street.

